# The Story of the Hospitals

**Published:** 1887-05-21

**Authors:** 


### The Story of the Hospitals.

### The London Hospital.

(*Concluded from vol. i, p.* 158.)

**A Quaint Story.** In a previous article I referred briefly to the great reform in the out-patient department which was inaugurated in 1884. A somewhat curious circumstance first caused the house-governor to turn his attention to this important but very difficult question. Formerly the out-patients' tickets had one side left blank for prescriptions, and the patients used to take away with them, after each visit, these same tickets as their passes for admission at every subsequent visit. One day the house-governor—who is ever keeping keen watch and ward over his institution—was noticing the stamping of the date on these tickets, when presented as admission passes. Among the patients was an Irishwoman. The porter, after scrutinising the good woman's ticket, demanded of her, "Are you Terence O'Reilly?" "Shure, no, by the powers, bedad," responded the Hibernian dame; "my name, to be shure, is Bridget McCarthy." "Then why did you bring this ticket with you?" continued the porter. "Shure," answered Bridget, "an' it was on the lodging-house table with wan or two ithers, an', anyhow, it's the blessed physic as 'ill do me a power o' good." But the porter was inexorable. The good woman had to go home again and bring her right ticket. It was just as well that the porter did insist on this being done, for when Bridget made her second appearance, it was found that the ticket contained a prescription for some internal malady, while Terence O'Reilly was the unfortunate victim of a sore leg, for which some special ointment had been prescribed. An ointment suitable for a sore leg might result in disagreeable consequences when taken internally, and Bridget, if she had taken it and survived to tell the tale, would no doubt have chosen an adjective somewhat antithetical in meaning to "blessed," in order to express her convictions on the point.

**The System Reformed.** Small occurrences have sometimes been fruitful in the attainment of great ends. The fall of the apple, as Newton sat in his garden at Woolsthorpe—if we believe the story—resulted in the marvellous revelation to science of the discovery of the law of gravitation. Now, Bridget's mishap disclosed to the house-governor of the London Hospital a grave flaw in the old order of things, and so he set to work to remodel the whole system of out-patients' relief. The *modus operandi* which Mr. Nixon devised constitutes a vast change for the better. Under the new *régime* the patient is furnished with a reference-card, which serves as his pass till cancelled, the ticket and prescription being held in a book-cover, which has a number corresponding to that on the card. These books are all taken charge of when presented at the dispensary window, and are then re-sorted and lodged in special receptacles, standing in the out-patients' waiting-hall. At the present moment there are, I believe, no less than 10,000 books of patients in attendance.

**The Maternity Department.** The out-door maternity department claims from me, before I pass on, a few words of notice. It was established in 1854, and is intended primarily for the benefit of students of midwifery. Owing to the great increase in the population, only poor married women resident within a one-mile radius of the hospital are admitted; but, with this limitation, the cases are far too numerous for anything like all to receive the attention of the students. Last year the students attended over 2,700 cases at the homes of the women. The parochial authorities, unfortunately, take an undue advantage of this department, which is, as I have said, intended to be educational only.

**In the Cellars.** The laboratory—the great manufactory of physic—is a wonderful department. Everything seems to be provided for making things on a huge scale. An exploration of the cellars, which have been excavated and made, only increases our astonishment. In one we come upon the wines and spirits, and in another upon the acids and poisons, which are isolated for safety. One monster tank, we are told, contains cod-liver oil, and another lard. The dispenser's annual return of the cost of drugs, chemicals, and other hospital sundries, is an interesting and instructive document, upon which I hope on a future occasion to be able to touch more fully. Leeches have almost disappeared from it. In 1885 they cost only £3 4*s.* 11*d.*, while in 1839 they figured at £495! Then bleeding was a practice in high vogue. I learnt from the dispenser that the hospital makes all its own soda-water. If it were bought, it would cost something like £600 per annum. Making it at the hospital, the labour and chemicals average about £50 a year, and, allowing for wear and tear of materials, the saving effected may certainly be put down at £500. Nearly three tons of ice a week are used at the London Hospital.

**The Kitchen.** The kitchen at the London Hospital should be visited about noon, just when the dinner is on the point of being served. It is a fairly large apartment, beautifully clean and cool, for all the cooking is done by gas and steam. Formerly, the cooking was done by coal fires, and the substitution of Leoni's gas-apparatus has, I believe, resulted in a very material saving. When I remind my readers that, patients and officials together, nearly a thousand mouths have to be fed at the London every day, they will perhaps be able to form some slight idea of the culinary operations. Here are a few of the annual provision-bills:—Milk, £2,600; eggs, £820; vegetables, £800; butter, £650; bread, £1,400; meat, £5,500. The water costs about £200 a year, and gas and coal about £2,700.

**In the Gloucester Ward.** As I entered the kitchen, I learnt that the dinner was "just on," and after seeing one section of it despatched up the lift, I hurried with the house-governor to meet it at the Gloucester Ward. This is a large, excellently arranged apartment for men. The blue-washed ward seemed very light and cheerful. The bright coverlets of the beds, and the flowers on the table at sundry intervals, made it look quite lively. We met the dinner, and followed it into the ward, where Sister Gloucester, with an attendant group of nurses, was busily engaged in the process of carving out and weighing the requisite portions. "The flowers look very pretty," I observed to the house-governor. "Yes," he replied; "we get immense quantities sent by friends—cab-loads, sometimes waggon-loads; the Bible Flower Mission send once a week. Every ward is liberally supplied."

**In the Casualty Hall.** An inexperienced visitor to the Casualty Hall would be inclined to fancy that, by some untoward circumstance, everybody must that morning have met with an accident. Here minor casualties or serious accidents are received day and night throughout the year. The accidents last year treated as "out-patients" numbered over 6,000, while the minor casualties amounted to nearly 32,000. During the twenty-four hours just prior to my visit, I learnt that no less than 563 cases had been attended to. At night, two porters and a nurse are left in charge. I must confess that I was somewhat surprised at the very limited dimensions of the apartments where the casualties, male and female, are seen. Surely some improvement might be made in this respect.

**The Laundry.** The laundry is another interesting apartment. Some 7,000 pieces—the sheets alone number 3,000—go to the "wash." There is a private laundry attached, where the officers' linen is washed. The washing is all done by Bradford's washing machinery.

**The Dead-house.** Leaving the laundry, I next paid a visit to some essential, but less agreeable premises. The house-governor having shown me the room where the Coroner holds his inquests, I passed on to the depository for the dead. Before me I saw several suspicious-looking little doors; and on my right some bundles. "Do you smell anything at all?" asked my conductor of me. I was compelled to answer in the negative; for though I knew I was in the apartment where the dead lie pending removal, my olfactory nerves detected nothing in the least offensive. After glancing at an ominous little list, the house-governor added, "There are now seven bodies here," reading me out the names, and, he continued, "those bundles are the clothes waiting for the friends."

**About the Nurses.** The nursing at the London is now on a very satisfactory footing. In 1873 the first attempt was made to organise a system of training nurses for the special service of this hospital. First this was attempted out of doors, but the inconvenience of this plan soon made itself apparent. In December 1874 suitable accommodation was provided at the hospital, and a training-home for sisters and nurses now forms part of this great establishment. I went over this section of the hospital, and was very pleased to see the comfortable quarters provided. The nurses off duty have a very cosy sitting-room. The dining-room, where the nurses dine in batches of about forty, is a large and admirably arranged room. The sisters, of course, dine separately from the nurses. The bedrooms of the nurses are neatly and well furnished, each possessing every necessary requisite. Miss Lückes, the matron, is herself a highly experienced lady, and the nurses under her management are well cared for.

**The Hebrew Ward.** The London Hospital possesses a distinctive feature in a Jewish ward. The importance of such special accommodation is evident, when I mention that the yearly average of Hebrew patients treated in this special ward amounts to nearly 700. Mrs. Lynn Linton gives the following vivid description of this interesting feature of the London Hospital:—"In the Jews' ward we come upon a different physiognomy, and certain differences of condition. To begin with, the poorer Israelites have all a German's horror of ventilation, and draw their bed-curtains close about their heads and faces whenever they have the chance. They have all things separate and appropriate; their own meat killed according to the law by their own butcher; the two distinct waste-pipes, by which their own kitchen-refuse is carried away, so that the washings of the plates and dishes where meat has been may not be mingled with other articles of food; during Passover, separate milk; and a special cupboard where all utensils used during Passover are kept, and never touched, nor even looked at in the interim. The cupboard might swarm with mice or cockroaches, red ants or blackbeetles, but it would not be opened even for the purposes of cleansing, so strict are these ancient people in the smallest as well as the greatest matters of their faith. A piece of Passover-cake, renewed yearly, is fastened over the door of the ward appropriated to them, and a scroll of the law, in a small tin cylinder, remains as the sign and shield of the race which it both demonstrates and protects.

